# Effect of direct reciprocity and network structure on continuing prosperity of social networking services

**DOI:** 10.1186/s40649-017-0038-2

**Published:** 2017-05-26

**Authors:** Kengo Osaka, Fujio Toriumi, Toshihauru Sugawara

**Affiliations:** 1 Department of Computer Science and Communications Engineering, Waseda University, 3-4-1 Okubo, Shinjuku, Tokyo, 169-8555 Japan; 2 Graduate School of Engineering, The University of Tokyo, 7-3-1 Hongo, Bunkyo, Tokyo, 113-8656 Japan

**Keywords:** Social networking services, Meta-norms game, Reciprocity, Evolutionary game, Complex networks

## Abstract

**Background:**

Social networking services (SNSs) are widely used as communicative tools for a variety of purposes. SNSs rely on the users’ individual activities associated with some cost and effort, and thus it is not known why users voluntarily continue to participate in SNSs. Because the structures of SNSs are similar to that of the public goods (PG) game, some studies have focused on why voluntary activities emerge as an optimal strategy by modifying the PG game. However, their models do not include direct reciprocity between users, even though reciprocity is a key mechanism that evolves and sustains cooperation in human society.

**Proposed methods:**

We developed an abstract SNS model called the reciprocity rewards and meta-rewards games that include direct reciprocity by extending the existing models. Then, we investigated how direct reciprocity in an SNS facilitates cooperation that corresponds to participation in SNS by posting articles and comments and how the structure of the networks of users exerts an influence on the strategies of users using the reciprocity rewards game.

**Experimental results:**

We run reciprocity rewards games on various complex networks and an instance network of Facebook and found that two types of stable cooperation emerged. First, reciprocity slightly improves the rate of cooperation in complete graphs but the improvement is insignificant because of the instability of cooperation. However, this instability can be avoided by making two assumptions: high degree of fun, i.e. articles are read with high probability, and different attitudes to reciprocal and non-reciprocal agents. We then propose the concept of half free riders to explain what strategy sustains cooperation-dominant situations. Second, we indicate that a certain WS network structure affects users’ optimal strategy and facilitates stable cooperation without any extra assumptions. We give a detailed analysis of the different characteristics of the two types of cooperation-dominant situations and the effect of the memory of reciprocal agents on cooperation.

## Background

Social networking services (SNSs), such as Twitter, Facebook, and Google+, are frequently used by many users around the world not only to share and exchange local information among limited specialized and close-friend groups but also to publish/obtain public information for the purposes of exchanging opinion, advertising, marketing, and politics [1, 2]. SNSs are usually run by companies and non-profit organizations but cannot persist without huge amounts of up-to-date content being continually posted by individual users. Such user activities incur various costs in terms of creating and submitting the content, and why users continue to post articles and comments is not well understood. In addition, *free riders* (or *lurkers*) exist, that is, users who just read the content and never post articles. To provide incentives to individual users to keep submitting content, many SNSs have introduced specific mechanisms, such as providing comments on articles, comments on comments, indication of the number of followers, signs showing articles have been read, and “Like” buttons. These mechanisms can provide quantitative rewards (e.g. showing the numbers of readers of articles and users who clicked on the “Like” buttons) as well as psychological rewards that provide feelings of connection to people and a sense of belonging [3]. However, these incentive mechanisms also rely on users’ voluntary behavior and, thus, incur some cost and time.

As the variety of social media on the Internet continues to grow, it is becoming important to identify the conditions, mechanisms, and/or design methodologies inherent to maintaining an active and thriving SNS. Thus, a number of approaches, such as network analysis [4, 5], social psychology [6], analysis of exchange patterns [7], and evolutionary game theory [8], have been used to tackle this issue. Here, we focus on the evolutionary game theoretic approach since the mechanisms maintaining for thriving of SNSs have not been fully investigated from this viewpoint.

In this approach, SNSs are assumed to have the characteristics of a public goods game, because SNS are shared resources sustained by many users. For example, Toriumi et al. [8] and Hirahara et al. [9] modeled an SNS as a *rewards game* (RG) and a *meta-rewards game* (MRG), which are dual parts of Axelrod’s meta-norms game, and their own extension, called an *SNS-norms game*, to identify evolved behaviors of *agents* that model SNS users. They then analyzed the conditions under which a *cooperation-dominant situation* arises, where cooperation in this game corresponds to posting an article and a comment and a cooperation-dominant situation corresponds to a situation in which most users post them, so SNSs are active. They found that meta-rewards such as comments on article comments [8] and a simple (so, low-cost) response mechanism for rewards such as “Like” buttons for articles [10] play an important role in SNSs. However, these studies did not consider social and personal relationships between peers. Furthermore, some SNSs have no mechanism to provide meta-rewards, so another mechanism and/or interactive structure also seems to affect SNS activities.

Cooperation is a key activity to maintain public goods games. Nowak [11] pointed out that at least one of five mechanisms—kin selection, direct and indirect reciprocity, network reciprocity, and group selection—is necessary for cooperation to evolve in human society. Rand and Nowak [12] subsequently showed empirical evidence of human cooperation sustained by these mechanisms. Other studies have also reported that these mechanisms, especially reciprocity, exist and that reciprocity plays a crucial role in online networks [7, 13, 14]. We believe that reciprocity, especially direct reciprocity, is essential in an SNS because connections between users are usually established through direct interaction such as “reading articles” and “commenting on articles”.

Thus, our study was aimed at investigating the effect on direct reciprocity between users on cooperation-dominant situations and how network structures affect the users’ interaction strategy. For this purpose, we extended an existing abstract model of an SNS [8] to include direct reciprocity. The extended model is called a *reciprocity (meta-)rewards game* whose structure is similar to the (M)RG, but agents tag peer agents and decide their behaviors on the basis of recent reciprocal behaviors of these peers. We then tried to determine the circumstances in which the rates of cooperation increase and when the established cooperation collapses in this game. Note that we particularly focused on the reciprocity rewards game that has no mechanism for giving a meta-reward because a number of thriving SNSs do not have meta-rewards as mentioned above; thereby, we wanted to find another mechanism or interaction structure that promotes a thriving SNS.

We experimentally show that in complete graphs, the Watts–Strogatz (WS) model [15], the connecting nearest neighbor CNN model [16], and Barabasi–Albert (BA) model [17] networks, users do not cooperate with all neighbor agents but rather with a few close friends established on the basis of past reciprocal behavior. To explain this phenomenon, we propose the concept of *half free riders* (or *partial lurkers*) and discuss the interaction structure needed to maintain a cooperation-dominant situation in which the SNS continues to prosper. This is one main difference from the previous studies [8, 10]. Our experimental results suggest that user behavior like a half free rider sustains cooperative activity in all types of network. However, this phenomenon raises in complete graphs under the additional conditions that (1) agents seldom miss the posted articles and (2) they do not comment on articles posted by non-reciprocal agents. We also conducted experiments using WS, BA, and CNN networks and tried to understand how the network structure affected reciprocity, and thus the prosperity of the SNS. We found that the network structure strongly affects continuation of a cooperation-dominant situation without any additional conditions. We also examined the (non-)reciprocal relationships and the effect of varying the term of memory of the reciprocal agents.

## Related work

A number of studies have attempted to understand what factors affect social media by analyzing the network structures of social media. For example, Karamon et al. [4] devised an algorithm that can analyze important network-based features of huge social networks for better understanding the user behavior therein. Saito and Matsuda [5] analyzed network structures to identify two key types of user who draw the attention of many other users on Twitter and showed that one type has higher link reciprocity. In the field of social psychology, Lin and Lu [6] empirically studied reasons people have for joining SNSs and found that enjoyment is the most influential factor for people to continue using an SNS. They also found a notable difference between genders, i.e. the number of active peers is an influential factor for women’s enjoyment, and results in their continued use of social media, whereas the number of members has an insignificant effect on men’s enjoyment. Qasem et al. [2] attempted to detect influential actors in SNSs in order to identify who can influence others and to improve information diffusion and market efficiency.

A number of studies have paid attention to reciprocity between users by conducting empirical analyses. For example, Surma [18] focused on reciprocity because it is crucial in social exchanges. He analyzed reciprocity on Facebook and showed strong empirical evidence that reciprocity messages sent from a user on online social networks increase reciprocity reactions from her/his audience. Faraj and Johnson [7] found that network exchange patterns in an online community are characterized by reciprocity patterns and are different from those characterized by preferential attachment [17]. Takano et al. [14] analyzed player action logs and found that cooperation based on reciprocity could be observed in a network game. These papers suggest that reciprocity is a key factor in keeping social media thriving.

A number of studies proposed abstract models of social media and investigated their properties. Anderson [19] proposed a game theoretic approach aimed at understanding how social media emerge as a driving force in contemporary marketing and how this would affect future marketing. Toriumi et al. [8] revealed that SNSs have similar properties to public goods games, but they correspond to the dual part of the meta-norms game [20] because SNSs seem to lack a means of punishing non-cooperators and only give (psychological) rewards to cooperators. Their model includes two games, the rewards game (RG) and the meta-rewards game (MRG), and they indicated that meta-rewards facilitate cooperation, resulting in active use of an SNS. Hirahara et al. [9, 10] subsequently extended this model to the SNS-norms game that includes the characteristics of the interaction patterns in SNSs. They ran this game in a variety of complex networks and found that users at network hubs facilitate posting articles to some degree even if no meta-reward is provided. However, their study did not take into account reciprocal relationships between peer agents.

We have already presented a model including reciprocity in an SNS [21], but described only a few experimental results on it. This paper is an extended version of our previous workshop paper [21], and it details more thorough experiments using a variety of complex networks and discusses the characteristics of our model through a detailed analysis.

## Proposed model for social networking services

### Reciprocity rewards and meta-rewards games

**Fig. 1:**
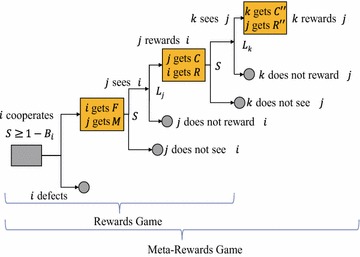
Meta-rewards and rewards games

**Fig. 2:**
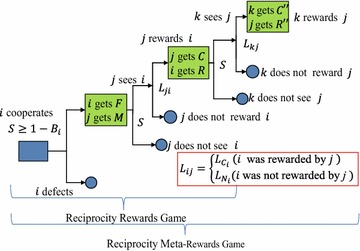
Reciprocity (meta-)rewards game

Social networking services are sustainable only when many articles and comments on them are posted by and shared among many anonymous participants. Although some cost personal time and effort, users can obtain information by reading such posts and can receive responses that provide feelings of connectivity, empathy, and contentment. On the other hand, there are many free riders who only read content. Therefore, SNSs have the properties of public goods that are produced and sustained by continuous cooperative activities in the SNS community. The game structure of this mutual interference is essentially an *n*-person prisoner’s dilemma (PD) game. Toriumi et al. [8] proposed RG and MRG as dual games of the norms and meta-norms games proposed by Axelrod [20] (Fig. 1) and attempted to explain the mechanism of continuous voluntary participation in SNSs. Although they showed that a meta-reward, which corresponds to “comments on a comment” in an SNS, for example, can provide an incentive to a user for voluntary participation, they ignored reciprocity, which is crucial to characterizing activities in SNSs [7, 14, 18]. Hence, we devised the *reciprocity rewards game* (RRG) and the *reciprocity meta-rewards game* (RMRG) by incorporating reciprocal relationships among agents into the RG and MRG (see Fig. 2).

Let $A=\{1, \ldots, \,n\}$ be the set of agents. Agents are connected with a graph $G=(A,E)$, where *E* is the set of links between agents. The set of neighbor agents of $i\in A$ is denoted by $A_i$ (${\subset} {A}$). Agents in an R(M)RG game select the strategy of either *cooperation* or *defection*. Cooperation indicates posting articles and comments, and defection indicates just reading them. A user who almost always selects defection is defined as a *free rider*. Agent $i\in A$ has three learning parameters: the probability of cooperation (i.e. posting a new article) $B_i$, the probability of giving rewards (e.g. posting a comment on the article) to reciprocal agents $L_{C_i}$, and the probability of giving rewards to other (normal) agents $L_{N_i}$. We call $B_i$, $L_{C_i}$, and $L_{N_i}$ the *article posting rate*, the *reciprocal comment rate*, and the *normal comment rate*, respectively. We also call $L_{C_i}$ and $L_{N_i}$ the *comment rates* hereafter. To apply the *genetic algorithm* (GA), we express each of these parameters as three bits, so they take on a discrete, i.e. value 0/7, 1/7,…, or 7/7. This expression is identical to the one used in the meta-norms game [20]. Agent *i* has a *memory for reciprocal agents* $W_i$ (${\subset} {A_i}$), which is the set of reciprocal agents that are defined as neighbor agents that posted comments on *i*’s articles in the most recent $T_{\rm{W}} ({>}{0})$ rounds. Positive integer $T_{\rm{W}}$ is called the *memory term*. We define a *round* as the period in which all agents perform R(M)RG or (M)RG once with the neighbor agents.

An RRG or RMRG proceeds as follows. For $\forall i\in A$, the parameter $S_i^t$ ($0\le S_i^t\le 1$) is defined randomly or with a certain method in the *t*-th round (*t* is a positive integer) when *i* is going to post an article. Intuitively, $S_i^t$ expresses the degree of fun of (or the degree of attention to) the contents of the article that *i* is going to post and the associated comments after that. Thus, if $S_i^t \ge 1-B_i$, *i* posts a new article with cost *F* and agent $\forall j\in A_i$ reads the article posted by *i* and gains reward *M* by reading it. Then, *j* proceeds to the next phase with probability $S_i^t$. Then, agent *j* comments on the article with probability $L_{ji}$, where $L_{ji}=L_{C_j}$ if $i\in W_j$; otherwise $L_{ji}=L_{N_j}$. Then, *j* pays a cost *C*, and *i* gains a reward *R* through *j*’s comment. The game chain so far is referred to as the RRG.

Subsequent to the RRG, $k\in A_i$ reads *j*’s comment and proceeds to the next phase with probability $S_i^t$. If this happens, *k* posts a response to the comment with probability $L_{kj}$, where $L_{kj}=L_{C_k}$ if $j\in W_k$ and $L_{kj}=L_{N_k}$ if $j\not \in W_k$. When *k* posts it, *k* pays a cost $C''$ and *j* gains a reward $R''$. The RMRG ends here. Note that, because reciprocity is not taken into account in RG or MRG, agent *i* has only two parameters, $B_i$ and $L_{N_i}$ ($L_{N_i}$ is denoted by $L_i$ in RG) and $W_k=\emptyset$. $L_i$ in (M)RG is called the *comment rate* after this. The glossary of variables used in the RRG and RMRG is shown in Table 1.

From the definition of reciprocal agents, a reciprocal relationship is mutual or one sided, and expresses the internal cognitive state of each agent. We think that the two types of reciprocity exist in the human relationships and thus in SNSs. This will be discussed using the experimental results in the "Analysis of reciprocal relationship" section.

**Table 1:** Parameters/variables, descriptions in RRG and RMRG, and values in the experiments below

Parameter	Description	Value
*A*	Set of agents	$|A|=20\;{\text{or}}\;1000$
$B_i$	*i*’s probability of posting an article	Variable
$L_{C_i}$	*i*’s probability of posting a comment to a reciprocal agent	Variable
$L_{N_i}$	*i*’s probability of posting a comment to a normal agent	Variable
$S^t_i$	Degree of fun of *i*’ article	$0<S^t_i <1$ (random)
*F*	Cost of posting article	$-3.0$
*M*	Reward for reading article	1.0
*C*	Cost of comment	$-2.0$
*R*	Reward for receiving comment	9.0
$W_i$	*i*’ memory of reciprocal agents	Variable
$T_{\rm{W}}$	Memory term	1 (if nothing mentioned)

### Evolution by genetic algorithm

Reciprocity rewards game and RMRG are evolutionary games, as are the (meta-)norms game and (M)RG, and we define the evolutionary parameter setting in accordance with their experiments [8, 20]. One generation of the game is defined as a period in which all agents have four chances to post articles and write/receive comments related to them.^1^ At the end of one generation, each agent selects two agents as parents from its neighbors on the basis of *fitness values*, which are defined as the cumulative rewards received minus the cumulative costs incurred during the current generation. This process is continued up to a certain generation.

Each of three learning parameters, $B_i$, $L_{C_i}$, and $L_{N_i}$, is represented as a three-bit gene, and each agent has a nine-bit gene.^2^ The initial values of the nine-bit genes are set randomly. The evolution consists of three phases: (1) parent selection, (2) crossover, and (3) mutation. A child agent of *i* for the next generation is generated as follows. First, in the parent selection phase, *i* selects two parent agents from *i* and *i*’s neighbor agents on the basis of a probability distribution $\{\Pi _j\ |\ j\in A_i\cup \{i\}\}$ defined as

1 $$\begin{aligned} \Pi _j= {(v_j - v_{\text{min}})^2}/{\sum _{k\in A_i\cup \{i\}} (v_k - v_{\text{min}})^2}, \end{aligned}$$

where $v_k$ is the fitness value of agent $k\in A$, and $v_{\text{min}} = \min _{i\in A_i\cup \{i\}} v_i.$ Then, two new genes are generated using uniform crossover from the genes in the selected parent agents and one of them is randomly selected in the crossover phase. In the mutation phase, each bit of the gene of the child agent is inverted with a probability of 0.005. This means that if there are 20 agents in the network, 0.9 bits will mutate on average. After that, the derived gene is used for the child agent of *i*.

## Experiments and discussion

### Experimental setting

We focused on the reciprocity rewards game (RRG) in the experiments because the reward had by commenting on a comment seems small and thereby insignificant in an SNS. Furthermore, simple response mechanisms (rewarding mechanisms), such as “Like” buttons and “read” icons, cannot be used to give meta-rewards for these simple responses. In the first experiment (Exp. 1), we compared the results of the RRG with those of the RG [8] and investigated the features of the RRG. The agent network (*A*,*E*) was a complete graph; we chose it because it was used in Toriumi [8] and also seems to be a basis of comparison with those when (*A*,*E*) are other types of network. The purpose of Exp. 1 was to investigate how reciprocity affected user behavior in an SNS by comparing the transitions of the average rates of cooperation, that is, posting an article or a comment, in the RG or RRG. Then, we also tried to ascertain the causes of sustainability and collapse of cooperation in the RG and RRG, as well as the reciprocal interaction structure in the RRG. The second experiment (Exp. 2) investigated how network structures affect cooperation and the reciprocal structure in RRG. For this purpose, we ran RRGs on the WS, CNN, and BA networks and compared their results with those of complete graphs. In the third experiment (Exp. 3), we clarified the effect of the memory term, $T_{\rm{W}}$, on the evolution of cooperation in various networks. Note that the experimental data described below are the average values of 100 independent experimental runs based on the different random seeds.

**Table 2:** Glossary of parameters defined in the "Experiment and discussion" section

Parameter	Description
*B*	Average of $B_i$ in RG and RRG
$L_C$	Average of $L_{C_i}$ in RRG
$L_N$	Average of $L_{N_i}$ in RRG
*L*	Average of $L_i$ in RG
$S'$	Fixed value of the degree of fun of articles
$L_{N_{\rm{max}}}$	Maximum value of $L_{N_i}$
*p*	Re-wiring probability in the WS model
*u*	Conversion probability in the CNN model
*m*	Number of links when a node is added in the BA model
$r^a$	Average number of reciprocal agents, $r^a_i$
$r^m$	Average number of mutual reciprocal agents, $r^m_i$
$r^o$	Average number of one-sided reciprocal agents, $r^o_i$

### Role of reciprocity on cooperation—complete graphs

**Fig. 3:**
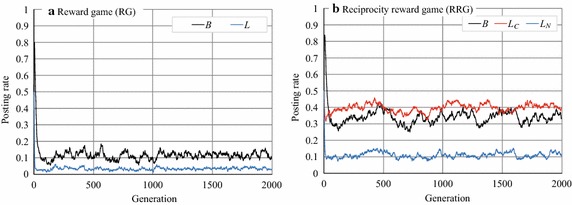
Article posting and comment rates in RG and RRG

The parameter values in Exp. 1 are listed in Table 1. These values were determined on the basis of the experiments of Axelrod [20] and Toriumi et al. [8]. With this setting, Toriumi et al. [8] found that if the reward of comments was larger than the cost of posting articles and comments, cooperation emerged, but only in the MRG, and cooperation did not emerge if there was no meta-reward mechanism. Figure 3 indicates the probabilities of posting articles and comments over the generations. Note that we defined the average posting article rate as $B = \sum _{i\in A} B_i/|A|$, the average reciprocal comment rate as $L_C = \sum _{i\in A} L_{C_i}/|A|$, and the average normal comment rate as $L_N = \sum _{i\in A} L_{N_i}/|A|$ in the RRG. We also investigated *B* and the average comment rate $L=\sum _{i\in A}L_i/|A|$ in the RG. Note that the parameters that are and will be defined in this section are summarized in Table 2.

In RG on the complete graph (Fig. 3a), *B* and *L* undergo transitions at approximately 0.11 and 0.05, respectively; this result is consistent with that of Toriumi et al. [8] as mentioned above. On the other hand, in the RRG *B* and $L_C$ make transitions at approximately 0.33 and 0.40, respectively, and $L_N$ transitions at approximately 0.11 in RRG (Fig. 3b). These results indicate that the values of *B*, $L_C$, and $L_N$ are larger in the RRG than in the RG. These figures suggest that by taking into account reciprocity when deciding the behavior, the activity in the SNS improves, although the amount of improvement is quite limited. Another observation is that the dispersion of *B* and $L_C$ were large (Fig. 3b).

**Fig. 4:**
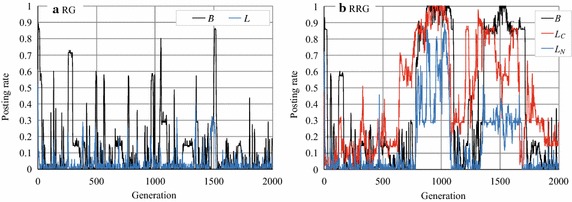
Article posting and comment rates in RG and RRG (an experimental run)

To see more clearly why *B*, $L_C$, and $L_N$ increased to a limited extent, we investigated one experimental run of RG and RRG (Fig. 4). Figure 4a indicates that the article posting and comment rates, *B* and *L*, in the RG, rose intermittently (but never reached 1.0) but then quickly dropped off. Such temporary cooperation was caused by mutation. However, the RG cannot maintain a cooperative situation; for example, when *B* and *L* increased in some agents, these agents temporarily gained rewards. However, because the free riders were benefited more in such a situation, cooperation soon disappeared.

On the other hand, in the RRG (Fig. 4b), *B* or $L_C$ sometimes reached (near) 1.0 and lasted much longer than in the RG (although they also dropped rapidly afterward). This means that almost all agents cooperate (by posting articles) and post comments on cooperators’ articles during this period. Furthermore, we can see that $L_C$ rarely fell to zero. The difference between the RG and RRG is that agents in the RRG distinguish reciprocal agents from other agents and so behave differently towards them. That is, agent *i* with a large $L_C$ comments only on articles posted by reciprocal agents who commented on past articles posted by agent *i*. Such selective comments prevent the collapse of cooperation by reducing the cumulative cost of making comments. However, they prevent collapse only when $L_C > L_N$; otherwise, when many agents begin to comment on arbitrary articles without enough rewards and free riders gain high total rewards, cooperation collapses like in the RG.

Now let us explain what the above phenomena correspond to in an actual SNS. When SNS users do not consider direct reciprocity (that is, they are in an RG), users who would otherwise often comment must stop commenting because the RG has no incentive for it and it is more beneficial for agents to behave as free riders. On the other hand, if individual users consider direct reciprocity when making comments, they preferentially comment on posts by reciprocal users preferentially by referring to their memory, $W_i$. Thus, when $L_C > L_N$, such selective comment behavior for receiving comments in the subsequent rounds sustains the norm for cooperation. We also believe that $L_C > L_N$ is a reasonable assumption in an actual SNS. We discuss this in the "Discussion" section.

### Sustainment or collapse of cooperation

We attempted to identify why the average rates of *B*, $L_C$, and $L_N$declined. The first factor that affects sustainability of cooperation is the relative values of $L_C$ and $L_N$, as mentioned above. Thus, we limited the maximum value of the *normal comment rate*, $L_{N_i},$ to $L_{N_{\rm{max}}}$ ($0\le L_{N_{\rm{max}}}\le 1$). Note that $L_{N_i}$ is expressed by three bits (it is a value ranging from 0 to 7), so the probability of the normal comment rate is $(L_{N_i}\times L_{N_{\rm{max}}})/7$. Another factor that affects the rewards of agents is the degree of fun of the posted articles and the associated comments, $S_i^t$. Thus, we set $S_i^t$ to a certain positive constant value $0\le S'\le 1$, and investigated how the evolution of cooperation changed as a result of varying $S'$.

**Fig. 5:**
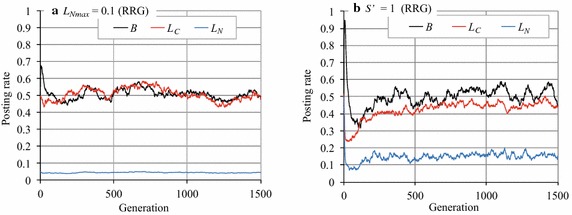
Article posting and comment rates in RRG ($L_{N_{\rm{max}}}=0.1$ or $S'=1.0$)

**Fig. 6:**
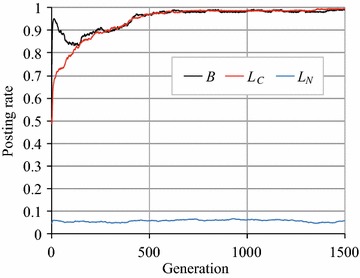
Article posting and comment rates in RRG ($L_{N_{\rm{max}}}=0.1$ and $S'=1.0$)

**Fig. 7:**
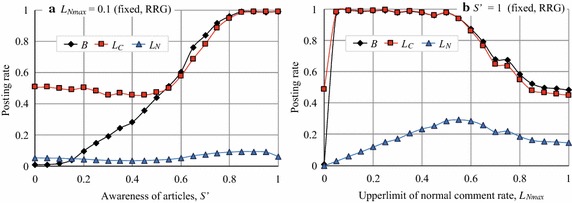
Change in article posting and comment rates in RRG

Figure 5 plots the changes in *B*, $L_C$, and $L_N$ over generations in the RRG when $L_{N_{\rm{max}}}$ was 0.1 (Fig. 5a) and when $S'$ was 1.0 (Fig. 5b). By comparing these figures with Fig. 3b, we can see that *B* and $L_C$ slightly increased in both cases, and no significant differences existed between them. However, when we set $L_{N_{\rm{max}}}= 0.1$ and $S'=1.0$, we observed the emergence of cooperation, as shown in Fig. 6. To investigate the influence of $L_{N_{\rm{max}}}$ and $S'$, we conducted a more detailed experiment. The results, plotted in Fig. 7, indicate how the posting rates varied in accordance with $S'$ ($L_{N_{max}}=0.1$, Fig. 7a) and $L_{N_{\rm{max}}}$ ($S'=1.0$, Fig. 7b). Figure 7b shows that when $S'=1.0$, if $L_{N_{\rm{max}}}\le 0.55$, *B* was nearly 1.0, but it gradually decreased to around 0.5 as $L_{N_{\rm{max}}}$ increased from 0.5 to 1.0. Conversely, Fig. 7a indicates that when $L_{N_{max}}=0.1$, if $S'\ge 0.8$, *B* remained almost 1.0, but as $S'$ decreased from 0.8 to 0, *B* gradually reached zero. Note that we will denote the RRG manipulated with parameters $S'$ and $L_{N_{\rm{max}}}$ by RRG($S'$, $L_{N_{\rm{max}}}$), respectively, e.g. RRG (0.8, 0.1).

These results indicate that $L_{N_{\rm{max}}}$ and $S'$ strongly affect the emergence of cooperation in the RRG: a smaller $L_N$ corresponds to the situation where agents may read articles from non-reciprocal agents but do not comment on them that much. A larger $S'$ corresponds to the situation where agents rarely miss articles posted by agents. Thus, while agents received more rewards from non-reciprocal agents by behaving like free riders, they commented on the articles posted by reciprocal agents. Both conditions must be satisfied in order to remove the causes of collapse from the RRG.

### Effect of network structure

To investigate the effect of network structure on the emergence of cooperation in RRG, we ran the RRGs on WS networks [15] whose average degree was 20 (since the complete graph in Exp. 1 was 20), on CNN networks [16] and BA networks. We set the number of nodes (agents) to 1000 in each network. Note that we did not use $S'$ and $L_{N_{\rm{max}}}$ in the RRG, and so did not fix the degree of fun of posts, *S*, or limit the comment rates to the usual neighbors, $L_{N_i}$, in Exp. 2.

**Fig. 8:**
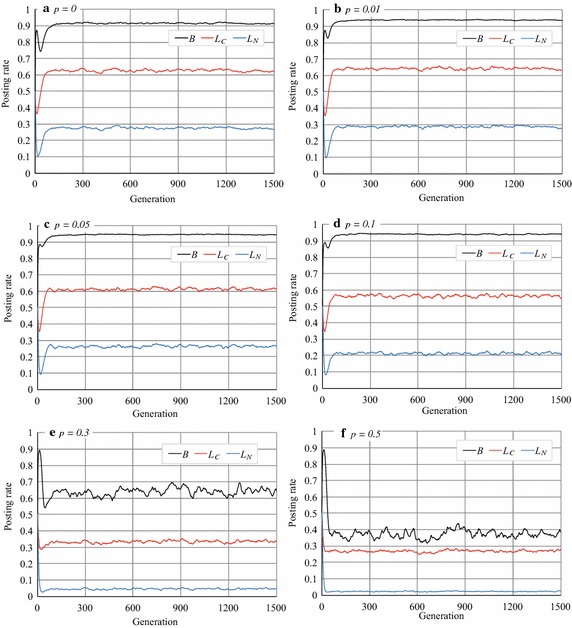
Article posting and comment rates in RRG (WS networks)

**Fig. 9:**
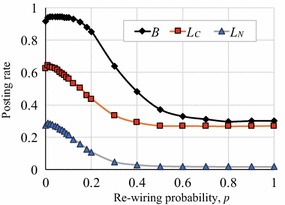
Article posting rates in WS network together with approximate polynomial curves

We observed quite different phenomena in the WS networks. As shown in Fig. 8, the rates of *B*, $L_C$, and $L_N$ remained relatively high and the dispersion of *B* was small when $0\le p\le 0.1$, where *p* is the *re-wiring probability* in the WS model. However, when $p=0.3$ and 0.5, *B* decreased and fluctuated more. Figure 9 shows how the average rates of *B*, $L_C$, and $L_N$ varied with *p* with which we can investigate the relationship between the re-wiring probability and the agent’s activity. Note that the WS model generates a regular graph when $p = 0$, whereas it generates a random network when $p = 1$ [22]. The cluster coefficients are small when $p>0.1$, so the small-world property with a large cluster coefficient only appears when $p\le 0.1$. Figure 9 indicates that *B* was around 0.94 when $p\le 0.1$, but as the re-wiring probability increased ($p>0.1$), cooperation became weak, and eventually *B* reached around 0.3, which was smaller than the *B* of the complete graphs (Fig. 3b). This result suggests that cooperation is dominant in WS networks with the small-world property and large-cluster coefficients. Note that *B* was small in the RG on the WS networks [23].

**Fig. 10:**
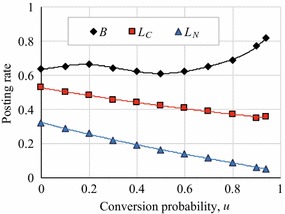
Article posting rates in CNN networks together with approximate polynomial curves

**Fig. 11:**
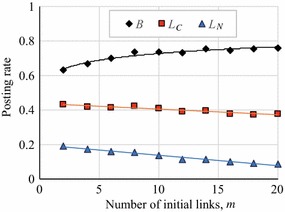
Article posting rates in BA networks together with approximate polynomial curves

Figure 10 shows how *B*, $L_C$ , and $L_D$ varied with the conversion probability, *u*, i.e. the probability for converting a potential link to a link [16] in the CNN model, and Fig. 11 shows the same in the BA networks in which we varied *m*, which is the number of links when a new node is added. These figures indicate that *B* ranged between 0.6 and 0.8, and gradually increased with *u* and *m*, although $L_C$ and $L_N$ decreased. The increase in *B* was due to the existence of hub agents with extremely high degrees: Even if $L_N$ is low, such hub agents receive comments from non-reciprocal agents. Thus, a strategy that makes $L_C$ and $L_N$ as small as possible is more optimal. However, such cooperation of hub agents was sustained by the neighbor agents in a non-cooperative strategy. We will discuss this phenomenon in the "Discussion" section.

### Stability/instability of agents’ strategies

**Fig. 12:**
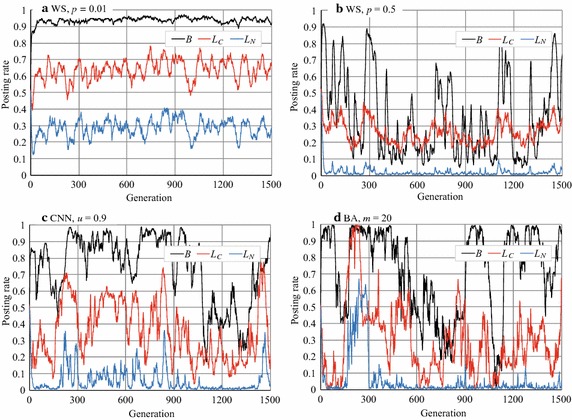
Article posting rates in WS, CNN, and BA networks (an experimental run)

The most different phenomenon observed in Exps. 1 and 2 was the dispersion of *B*. For example, *B* in Figs. 3b and 8e, f were fluctuated although they were the average values of 100 experimental runs. However, *B* was stable in Fig. 8a–d. The similar dispersion in *B* could be seen in CNN and BA networks whereas we do not show the graphs. To see the changes in the agents’ strategies, we randomly selected one experimental run and plotted the changes in posting rate (Fig. 12). In the RRG on the WS network with $p=0.01$, *B* was almost always larger than 0.9 and seemed stabler than in the other networks (Fig. 12a). However, on the WS network with $p=0.5$, *B* fluctuated with many sudden rises and falls (Fig. 12b). These sudden rises and falls appeared when *p* was around 0.2, and they gradually increased in height and numbers with *p*. Similar fluctuations appeared in the RRG on the CNN and BA networks (Fig. 12c, d) and in the RRG on the complete graph in Exp. 1 (Fig. 4b).

These data indicate that although the average values of *B* were comparatively a bit higher (around 0.7 and sometimes around 0.8) on the CNN and BA networks, as shown in Figs. 10 and 11, they were unstable; *B* was stable observed only on the WS networks with low *p* (${\le} {0.1}$) values. Although the similar stability was observed in the RRG (1.0, 0.1) in the complete graphs (Fig. 6), it had different characteristics; we will discuss this more in the "Discussion" section.

**Table 3:** Number of reciprocal agents

Network type	$r^a$	$r^m$	$r^o$
Complete graph (RRG)*	1.46	0.61	0.85
Complete graph RRG (0.8, 0.1)	4.65	3.66	0.99
Complete graph RRG (1, 0.1)	17.53	17.48	0.05
WS network ($p=0.01$, RRG)	3.70	1.41	2.29
WS network ($p=0.5$, RRG)*	0.34	0.07	0.27
CNN ($u=0.9$, RRG)*	0.72	0.23	0.49
BA ($m=10$, RRG)*	0.92	0.35	0.57
Facebook network (RRG)	4.30	1.55	2.75

### Analysis of reciprocal relationship

To express how many reciprocal neighbor agents and what types of reciprocal structure sustain cooperative behavior in agent networks, we should first introduce some notation. For agent $i \in A$ and for the set of *i*’s reciprocal neighbors $W_i$, we define the number of reciprocal neighbors as $r^a_i=|W_i|$. There are two types of reciprocity: $W^m_i = \{j\in W_i \ | \ i\in W_j\}$ is the set of mutual reciprocal agents of *i*, and $W^o_i = \{j\in W_i \ | \ i\not \in W_j\}$ is the set of one-sided reciprocal agents of *i*. If we express $r^m_i= |W^m_i|$ and $r^o_i= |W^o_i|$, $r^a_i = r^m_i + r^o_i$ obviously holds. Furthermore, we will express the average values of these parameters by eliminating the subscript *i*, e.g. $r^a = \sum _{i\in A}r^a_i$. Table 3 lists the relationship between the number of reciprocal agents and the network type. Note that the networks in this table have their average degrees equal or close to 20.

The average number of reciprocal agents, $r^a$, in the RRG was small on complete graphs, CNN networks, BA networks, and WS networks with high *p* values [marked with an asterisk (*) in Table 3]. Because $L_N$ was small, agents rarely received comments, and they eventually stopped posting. In this situation, a free rider was the optimal strategy. Nevertheless, *B* remained slightly higher than that on complete graphs (see the "Discussion" section).

On the other hand, the values of $r^a$ were around 4 in RRG (0.8, 0.1) on the complete graphs and in the RRG on the WS networks with a low re-wiring probability, *p*. These values were not large but large enough to maintain cooperation, i.e. posting articles by receiving comments. Of course, posting comments also incurred costs, but the agents behaved as free riders on the other non-reciprocal neighbor agents, and in so doing received sufficient rewards. We call such a strategy the *half free rider*, i.e. be cooperative with reciprocal agents, but otherwise behave as a free rider. Half free riders with appropriate numbers of reciprocal agents strike a balance between rewards and costs, and thus are optimal in these environments. A larger $r^a$ facilitates comments on articles, and, thus, it seems to sustain cooperation. However, we also have to consider the cost of commenting; we will discuss this aspect in the next subsection.

The characteristic that distinguishes the WS networks from the complete graphs is the ratio of $r^m$–$r^a$: Table 3 indicates that cooperation was sustained by mostly mutual reciprocal relationships in RRG (0.8, 0.1) and RRG (1.0, 0.1) on the complete graphs, but in the RRG on the WS network with $p=0.01$, it was sustained by both types of reciprocal relationship. Introduction of $S'$ and $L_{N_{\rm{max}}}$ generated mutual reciprocity, and RRG (1.0, 0.1) on the BA and CNN networks showed cooperative behaviors and many mutual reciprocal links (the graphs of this experiment are not shown). We will examine the meaning of $S'$ and $L_{N_{\rm{max}}}$ in an SNS in the "Discussion" section.

### Effect of memory term on cooperation

**Fig. 13:**
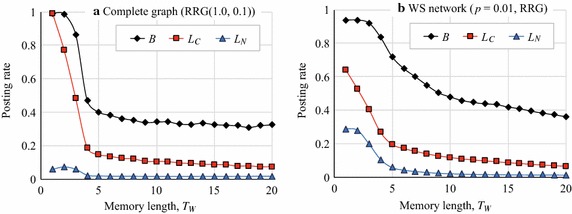
Relationship between memory term and article posting rates

In the third experiment (Exp. 3), we investigated how the memory term, $T_{\rm{W}}$, affected the agents’ behavior. Figure 13 plots the average posting rates, *B*, $L_C$, and $L_N$, when $T_{\rm{W}}$ was varied from 1 to 20. Here, we could intuitively say that cooperation should be facilitated because agents with a longer memory do not forget past reciprocal behavior. However, Fig. 13 indicates that in RRG (1.0, 0.1) on complete graphs (Fig. 13a) and in the RRG on the WS networks (Fig. 13b), a longer memory negatively affected the evolution of cooperation.

**Fig. 14:**
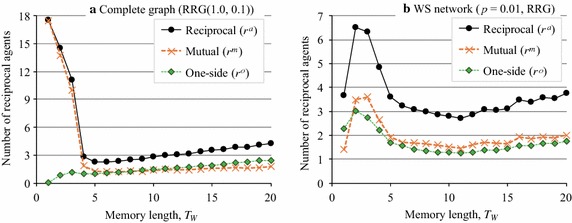
Relationship between memory term and number of reciprocal agents

We can think of two reasons for this phenomenon. First, when $T_{\rm{W}}$ is larger, agents do not forget the reciprocal behavior of neighbors, so they continue to comment on posted articles for a longer time. However, the opposite phenomenon also occurs. When $T_{\rm{W}}=1$, if agent *i* commented on an article of another agent *j* but *j* did not comment back on an article posted by *i*, *i* would eliminate *j* from $W_i$. However, when *i* has a longer memory, *i* would continue to consider *j* to be a reciprocal agent. Then, if *j* did not comment on *i*’s articles in a few rounds, *i* would still assume that *j* is a reciprocal agent. Second, agent *i* with a higher $r^a_i$ must engage in costly activity, because $L_{C_i} > L_{N_i}$ and *i* has to comment on more articles. Agents that did not comment on the posts of others but received comments would have an advantage. This situation continues for longer when $T_{\rm{W}}$ is large and, thus, is likely to lead to collapse of cooperation. Actually, Fig. 14a, b, which plots the average number of reciprocal agents versus $T_{\rm{W}}$, shows that the number of reciprocal agents decreased as $T_{\rm{W}}$ increased because of the increase in cost, although the larger $T_{\rm{W}}$ enabled agents to memorize more reciprocal actions by their neighbors.

If we compare Fig. 13a, b, we can see that the WS networks were somewhat robust to longer memory terms. The same phenomenon is apparent in Fig. 14a, b, which indicates that in RRG (1.0, 0.1) on the complete graphs, the number of reciprocal agents, $r^a,$ quickly decreased as $T_{\rm{W}}$ increased. On the other hand, in the RRG on the WS networks with $p=0.01$, $r^a$ increased when $T_{\rm{W}}=2, 3$, and 4 and cooperation was sustained by both mutual and one-sided reciprocal agents (Fig. 14b). However, the characteristics that were responsible for the robustness to memory in the WS networks are still unknown.

**Table 4:** Network characteristics of Facebook network

Network characteristics	Value
Number of users (agents)	4039
Average number of friends (degree)	43.691
Clustering coefficient	0.606
Characteristic path length	3.693
Power-law exponent	−1.180

### Games on a Facebook network

**Fig. 15:**
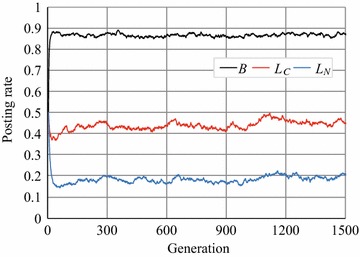
Average article posting rates (a Facebook network)

**Fig. 16:**
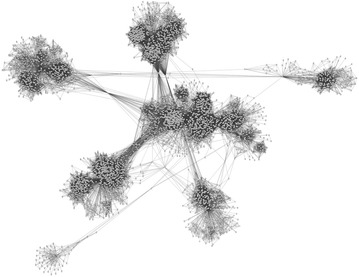
Facebook network structure

Finally, we conducted the same experiments using an instance of the Facebook network [24] whose features are listed in Table 4. Figure 15 plots the experimental results, while Table 3 lists the number of reciprocal agents in the RRG. Figure 15 indicates that the article posting rate *B* was around 0.88, which is close to that of the WS networks with *p* between 0.1 and 0.2, and that it was stable. In addition, the number of reciprocal agents, $r^a$, in Table 3 was around four and cooperation evolved and was sustained by both one-sided and mutual reciprocal agents, although $r^o$ was slightly larger than $r^m$, as in the WS networks. This instance of the Facebook network is more complicated than the networks used in Exp. 2, as is shown in Fig. 16, which visualizes it; it seems to consist of clusters of communities that have their own sub-structures and a few hub agents. However, our results suggest that the RRG on the Facebook network had characteristics similar to those of the RRG on the WS networks with* p* = 0.1–0.2, and cooperative behavior was sustained without introducing the parameters for manipulation, $S'$ and/or $L_{N_{\rm{max}}}$.

### Discussion

Here, let us discuss the experimental results from the viewpoint of an SNS. First, we can say that cooperation did not seem to emerge in the normal RRG on the complete graph, but if we manipulated RRG by introducing the parameters $S'$ and $L_{N_{\rm{max}}}$, cooperation (posting articles) emerged as shown in Figs. 6 and 7. If we look at these figures carefully, we see that *B* and $L_C$ had similar values, and this indicates that cooperation arose from $L_C$, i.e. high comment rates by reciprocal agents, mostly mutual reciprocal friends.

For example, cooperation emerged when $S'\ge 0.8$ and $L_{N_{\rm{max}}}=0.1$, i.e. RRG (0.8, 0.1). $L_{N_{\rm{max}}}=0.1$ means that users did not often comment on the articles if they were posted by non-reciprocal friends, while $S'=0.8$ means that users had attention to more than 80% of the posted articles by (reciprocal) users. We believe that this behavior is reasonable and frequently occurs in actual SNSs. Users behave as half free riders; they receive information (rewards) from non-reciprocal friends, but respond only to information from reciprocal close friends to sustain SNS (because the articles posted by close friends are likely to be interested). This activity balances rewards and costs in the SNS. Note that the phenomenon described above also occurred in the RRGs on the CNN and BA networks, that is, *B* and $L_C$ became about 1.0 when $S'\ge 0.8$ and $L_{N_{\rm{max}}}=0.1$.

On the other hand, even in a normal RRG, cooperation, i.e. posting articles and comments, became the optimal strategy on the WS networks with $p\le 0.1$ and the SNS seemed thriving. In this condition, WS networks have the properties of small-world and high cluster coefficient. Our experimental results also suggest that the instance of Facebook network that was generated in a bottom-up manner via interaction is likely to maintain cooperation. In addition, its average number of reciprocal agents was higher than those in other networks in the normal RRG. Although this network consists of a few communities, it has also the same properties (Table 4). Therefore, it is possible that these properties foster cooperation, but we need more study to clarify it.

Another implication from our experiments is that the number of reciprocal agents is important because the balance between rewards and costs because of reciprocal relationships strongly affects the agents’ strategies in a normal RRG. Now let us focus on the mutual and one-sided reciprocity in these networks. When $T_{\rm{W}}=1$, agents remembered the reciprocal behavior only in the next round, and the number of mutual reciprocal agents, $r^m$, was likely to be small, so $r^o > r^m$ in the normal RRG (Fig. 14b). However, when $T_{\rm{W}} \ge 2$, $r^m$ and $r^o$ had the similar values and they always coexist. This phenomenon was also true in the Facebook network. We believe that such a reciprocal structure is common in human society and, thus, observed in the SNSs in the real world. On the other hand, $r^m \gg r^o$ in RRG (1.0, 0.1) on the complete graph; thus, manipulation by $S'$ and $L_{N_{\rm{max}}}$ fostered cooperation by enhancing the effect of mutual reciprocity. This situation corresponds to a dense cluster that becomes active if all users are communicated closely each other.

On the other hand, Figs. 10 and 11 indicate that *B* in the normal RRG on the CNN and BA networks was larger than that on the complete graphs. This result reflects the existence of hub agents, i.e. agents that have extremely high degrees. Even on these networks, just reading articles (acting as a free rider) is optimal. However, a few agents comment on articles because of the mutation that corresponds to the vagaries of users. Thus, hub agents receive sufficient comments, have some reciprocal agents, and, thereby, continue posting articles. Because the hub agents earn rewards, their neighbor agents are likely to copy the hub’s gene for the next generation, and consequently *B* increases to some degree. However, this behavior is, of course, not optimal for non-hub agents; they cannot earn sufficient rewards. Hence, *B*, $L_C$ , and $L_N$ stopped increasing in the experiments. By contract, the WS networks have no hub agents. Thus, the essential cause of prosperity of the RRG resides in the network structure of the WS networks; this finding is not so obvious and its explanation remains an open problem.

## Conclusion

Social networking services are used by users around the world for purposes such as chatting with close friends, exchanging opinions, advertising, marketing, and politics. Although SNSs are usually run by companies and (non-)profit organizations, they depend on their users’ voluntary behaviors such as posting articles and comments. Since such voluntary behavior must incur some cost and effort on the part of the user, it is not obvious what makes them continue to participate in an SNS. Because SNSs have the features of the public goods game, a number of evolutionary game theoretic studies have attempted to understand the mechanism behind cooperation, which corresponds to posting articles in an SNS, by using extending the public goods game in various ways. However, conventional game theoretic models of SNSs ignore reciprocity, even though it plays an important role in evolving cooperation in human society. Thus, in this study, we devised a model, called the reciprocity rewards game (RRG), of an SNS that is based on the public goods game by incorporating direct reciprocity between users into the conventional model. After that, we ran the RRG on a variety of networks to see how reciprocity between users and the network structure affect cooperation. Our experimental results indicate that reciprocity in complete networks slightly raises the rate of cooperation, but the rise is not significant. However, if we manipulate the RRG so that users hardly miss any articles posted by their neighbors but at the same time hardly comment on articles posted by non-reciprocal agents, cooperation emerges. Second, in RRGs on WS networks with a low re-wiring probability *p*, cooperation emerges without any manipulation like in the RRG on the complete graph. We also analyzed the evolved reciprocal structures and the effect of the memory term on cooperation. Finally, using an instance network of Facebook, we conducted the same experiment, and its results indicate characteristics similar to those of WS networks with *p* between 0.1 and 0.2.

We found that a certain network structure facilitates cooperation in an RRG and makes cooperation robust to the memory term, but its essential cause is still unknown: this will be our future work. We also plan to add indirect reciprocity to our model and analyze the relationship between costs and rewards in continuous use of an SNS.

## Footnotes

1 The length of one generation is determined according to the previous studies [8, 20]. We also conducted our experiments in which the length of generation was set to six, eight, or ten as well as four, but we could not see a clear difference.

2 Likewise, two learning parameters in RG and MRG are encoded into a six-bit gene.
